# Apoptosis and Relevant Genes Are Engaged in the Response of *Apis mellifera* Larvae to *Ascosphaera apis* Invasion

**DOI:** 10.3390/insects16090925

**Published:** 2025-09-02

**Authors:** Tianze Zhang, Jingxian Li, Jiarun Yang, Xiaoxue Fan, Shiyu Mi, Xi Guo, Mengyuan Dai, Xihan Luo, Peiyuan Zou, Qingwei Tan, Dafu Chen, Jianfeng Qiu, Rui Guo

**Affiliations:** 1 College of Bee Science and Biomedicine, Fujian Agriculture and Forestry University, Fuzhou 350002, China; 13230693196@163.com (T.Z.); 18236228430@163.com (J.L.); 13064033836@163.com (J.Y.); imfanxx@163.com (X.F.); missy7728@163.com (S.M.); guo1136501019@163.com (X.G.); 18050348332@163.com (M.D.); 18206898837@163.com (X.L.); zoupeiyuan2216@163.com (P.Z.); hotony@163.com (Q.T.); dfchen826@fafu.edu.cn (D.C.); 2 National & Local United Engineering Laboratory of Natural Biotoxin, Fuzhou 350002, China; 3 Apitherapy Research Institute of Fujian Province, Fuzhou 350002, China

**Keywords:** apoptosis, *Apis mellifera*, *Ascosphaera apis*, TUNEL, survival, infection

## Abstract

Apoptosis is a genetically controlled process vital for homeostasis. This study examined the apoptotic response in the gut of *Apis mellifera* (*A. mellifera*) larvae to infection by *Ascosphaera apis* (*A. apis*) and its impact on host resistance and pathogen virulence. Here, Worker larvae of *A. mellifera* were inoculated with purified *A. apis* spores. We then quantified the expression of key apoptosis-related genes (*AmCaspase*-3, *AmBax*, and *AmBcl*-2) in the host gut and detected apoptotic cells via TUNEL assay. To functionally assess the role of apoptosis, larvae were treated with either the apoptosis inhibitor Z-VAD-FMK or the activator PAC-1, after which host survival, expression of apoptosis-associated genes, and the fungal virulence factor gene *Ste*11-*like* were analyzed. Our results showed that infection with *A. apis* significantly upregulated the expression of *AmCaspase*-3 and *AmBax* (*p* < 0.05) at 1–3 days post-inoculation (dpi), while the expression of *AmBcl*-2 was significantly reduced at 1 and 3 dpi (*p* < 0.05). Consistent with this, TUNEL assays revealed a markedly stronger green fluorescence signal in the guts of inoculated larvae at 3 dpi compared to uninfected controls, with clear co-localization of TUNEL and nuclear signals, confirming increased apoptosis. Pharmacological inhibition of apoptosis significantly enhanced the survival rate of *A. apis*-infected larvae, whereas apoptosis activation decreased larval survival. Accordingly, inhibiting apoptosis significantly suppressed the expression of *AmCaspase*-3 and *AmBax* (*p* < 0.001) and upregulated *AmBcl*-2 (*p* < 0.001). Conversely, apoptosis activation upregulated *AmCaspase*-3 (*p* > 0.05) and *AmBax* (*p* < 0.001), while significantly down-regulating *AmBcl*-2. Furthermore, apoptosis inhibition significantly down-regulated the fungal virulence gene *Ste*11-*like*, while its activation had the opposite effect. In summary, *A. apis* infection induces apoptosis in the larval gut by activating *AmCaspas*e-3 and *AmBax* and suppressing *AmBcl*-2. Inhibiting this apoptotic response enhanced host survival by modulating the expression of host apoptosis-related genes and the fungal *Ste*11-*like* virulence factor. These findings provide new insights into the host response to *A. apis* and suggest a potential strategy for controlling chalkbrood disease.

## Simple Summary

Honey bee larvae are threatened by a deadly fungus called chalkbrood, caused by *Ascosphaera apis* (*A. apis*). This disease kills honey bee larvae and hurts beekeeping. We wanted to understand if and how a natural process in the bee larvae’s gut, called apoptosis, helps defend against this fungus or makes things worse. We infected larvae with the fungus and looked at what happened to key cell death signals. We found that the fungus tricks the larvae into turning on too much cell death in their gut. Next, we tested two chemicals: one that stops cell death and one that forces it. Blocking cell death helped infected larvae survive much better and weakened the fungus. Forcing more cell death made survival worse and strengthened the fungus. Our results show that the fungus manipulates the larvae’s own cell death process against them to cause disease. Controlling this harmful cell death could be a new way to protect honey bees from this chalkbrood, helping save these important honey-producing insects and the crops they pollinate.

## 1. Introduction

Honeybees are critically important pollinators in global ecosystems, essential for maintaining biodiversity and supporting agricultural productivity. Among bee species, the Western honeybee (*Apis mellifera*, *A. mellifera*) is highly suitable for beekeeping due to its superior biological characteristics, and is therefore the most widely used species in commercial apiculture worldwide. It is valued for its contributions to ecological stability, scientific research, and economic development [1,2].

*Ascosphaera apis* (*A. apis*) is a filamentous fungus that infects honey bee larvae, causing chalkbrood disease. This lethal infection imposes substantial economic losses on the apicultural industry due to severe declines in colony population and productivity [3]. After spores are ingested by the larvae, they germinate within the gut lumen [4]. The mycelia then penetrate the peritrophic matrix and the gut wall, eventually breaching the body wall and proliferating across the integument. This process ultimately leads to larval death and the formation of a characteristic white, grey, or black mummified cadaver [5].

Apoptosis is a genetically programmed and highly conserved form of cell death that plays vital roles in development, tissue homeostasis, and host defense against pathogens in multicellular organisms [6]. This process is mediated by caspase-mediated protease cascades, which trigger characteristic biochemical and morphological changes such as cell shrinkage, chromatin condensation [7], DNA fragmentation, and the formation of apoptotic bodies [8]. These bodies are efficiently phagocytosed by neighboring cells or professional phagocytes, thereby avoiding inflammatory responses. In invertebrates, including insects, apoptosis serves not only as a key mechanism governing development but also as an essential component of the innate immune response against microbial infections.

A growing body of evidence indicates that filamentous fungal infections in insect hosts often dysregulate apoptotic pathways [9,10]. Infected hosts may initiate apoptosis in compromised or adjacent cells to restrict pathogen spread and nutrient acquisition [11]. Conversely, successful entomopathogenic fungi frequently employ molecular strategies to suppress, evade, or exploit host apoptosis, thereby promoting their own colonization, proliferation, and dissemination [12]. Such apoptotic modulation has been observed in multiple insect-pathogen systems, including the silkworm (*Bombyx mori*) [13], wax moth (*Galleria mellonella*) [14], cotton bollworm (*Helicoverpa armigera*) [15], and fruit fly (*Drosophila melanogaster*) [16], highlighting the complex role of apoptosis regulation in host–pathogen interactions.

Although apoptotic regulatory mechanisms have been elucidated in several model insects, the molecular basis of apoptosis in *A. mellifera*—particularly in response to *A. apis* infection—remains unexplored. Current research on honeybee apoptosis has primarily focused on infections by microsporidian pathogens (*Nosema* spp.), which suppress midgut *Caspase*-3 expression in midgut [17]. In contrast, *A. apis*, as an obligate tissue-invasive fungus, likely interacts with host apoptotic pathways in distinct and uncharacterized ways. Therefore, elucidating how *A. apis* influences apoptotic signaling in honeybee larvae will not advance our understanding of chalkbrood pathogenesis but may also uncover strategies used by the pathogen to evade host immune defenses.

This study investigated the apoptotic response in the gut of *A. mellifera* larvae following *A. apis* infection. Using pharmacological modulation of apoptosis—via inhibitors and activators—we assessed larval survival and the expression of key apoptosis-related genes. Our work aims to clarify the role of apoptosis as an innate immune defense mechanism during fungal infection in honeybee larvae. Furthermore, these results may inform novel pest management strategies that target apoptotic pathways and expand the understanding of agricultural insect immunology.

## 2. Materials and Methods

### 2.1. Bee Larvae and Fungal Spores

The *A. m. ligustica* worker larvae were derived from three colonies kept in the apiary of College of Bee Science and Biomedicine, Fujian Agriculture and Forestry University, Fuzhou city, China. Honeybee colonies exhibited no clinical symptoms of chalkbrood disease, and PCR assays returned negative results for *A. apis*.

According to the established technical procedures [18], preserved *A. apis* spores were inoculated onto Potato Dextrose Agar (PDA) solid medium for activation culture. Following incubation at 33 °C for 10 days in a biochemical incubator, black spore masses were observed covering the medium surface. Purification of *A. apis* spores was carried out as follows: In a laminar flow hood, surface hyphae were removed from sporangia by scraping. The sporangia were then transferred to tubes containing sterile water and vortexed. After centrifugation, the pellets were washed three times with sterile water. Purified spores, as confirmed by microscopy, were frozen in liquid nitrogen and stored at −80 °C. Spore suspensions were adjusted to working concentrations of 1.57 × 10^8^ mL^−1^ for infection or 1.57 × 10^5^ mL^−1^ for validation assays. *A. apis* was deposited in the China General Microbiological Culture Collection Center (CGMCC; Accession No. 40895).

### 2.2. Preparation of Gut Samples

Selected strong colonies provided a brood comb containing eggs and larvae, which was transferred to the laboratory. Purified spores were inoculated into 3-day-old larvae, divided into an inoculated treatment group (n = 48) and a non-inoculated control group (n = 48). Larvae were reared under laboratory conditions until they reached 4–6 days old. At 1–3 days post-inoculation (dpi), larval guts were extracted following a previously described method [19]. Specifically, midgut dissection was performed under sterile conditions in a laminar flow hood using sterilized microscissors and fine forceps.

### 2.3. TUNEL Assay

Midgut tissues from freshly collected 6-day-old *A. apis*-infected and uninfected larvae were immediately fixed in 4% paraformaldehyde for 24 h at 4 °C. The samples were then dehydrated through a graded ethanol series, cleared in xylene, and embedded in paraffin. Serial sections of 4–6 μm thickness were obtained using a microtome (Leica, Shanghai, China) and mounted on poly-L-lysine-coated slides. After deparaffinization and rehydration, sections were treated with 20 μg/mL proteinase K at 37 °C for 15 min to expose DNA fragmentation sites. Following three washes with PBS, sections were incubated with TUNEL reaction mixture (containing fluorescein-labeled dUTP and terminal deoxynucleotidyl transferase, TdT) in a humidified dark chamber at 37 °C for 1 h. Negative controls omitted the TdT enzyme. Nuclei were counterstained with DAPI for 5 min. Slides were mounted with anti-fade medium (ProLong™ Gold) and immediately examined under a fluorescence microscope (Nikon, Shanghai, China). All procedures were protected from light to maintain fluorescent signal stability.

### 2.4. Inhibition and Activation of Apoptosis

Three-day-old *A. m. ligustica* previously inoculated with *A. apis* were orally administered the apoptosis inhibitor Z-VAD-FMK or activator PAC-1 (5 μL/larva) at concentrations of 10 μM, 50 μM, or 100 μM. Larvae were divided into four groups per compound (three treatment groups + one control group, n = 20 larvae/group). Controls received 0.1% DMSO. Larvae were subsequently maintained on an artificial diet. Survival was monitored every 24 h, and survival curves were generated. The concentration demonstrating the most significant effect was selected as optimal. Optimal concentrations of activators and inhibitors were selected and administered to 3-day-old worker larvae via dietary supplementation. Midguts were dissected from 6-day-old larvae (n = 3 per treatment group) for further analysis.

### 2.5. Quantitative Real-Time PCR (qRT-PCR)

Total RNA was extracted from gut samples (n = 3 biological replicates) using the SteadyPure Quick RNA Extraction Kit (Accurate Biology, Hunan, China). cDNA was synthesized from total RNA using the Reverse Transcription Kit (Vazyme, Nanjing, China) and stored at −20 °C. Using qPCR, we quantified the expression of *AmCaspase*-3, *AmBax*, and *AmBcl*-2 genes, with *GAPDH* as the reference gene. Additionally, the relative expression of virulence factor genes was analyzed via qPCR using *A. apis Actin* as the reference gene [20]. Midguts from worker bee larvae treated with 0.1% DMSO were used as the control group.

The qPCR reaction mixture (20 μL total volume) contained 10 μL SYBR Green Mix (Yeasen, Shanghai, China), 1 μL each of forward and reverse primers (2.5 μmol/L), 1 μL of cDNA template, and 7 μL of DEPC-treated water. The primer sequences used are listed in Table 1. The thermal cycling protocol was as follows: initial denaturation at 95 °C for 3 min; 45 cycles (or 50 cycles for *A. apis* virulence factor detection) of denaturation at 95 °C for 15 s, annealing at 55 °C for 30 s, and extension at 72 °C for 15 s. Each sample was analyzed with three biological replicates, each comprising three technical replicates. Gene expression was quantified using the 2^−ΔΔCT^ method, and statistical significance was determined by one-way ANOVA in GraphPad Prism 8, with results presented graphically.

## 3. Results

### 3.1. A. apis Infection Induces the Apoptosis of the A. mellifera Worker Larval Gut Cells

**Figure 1:**
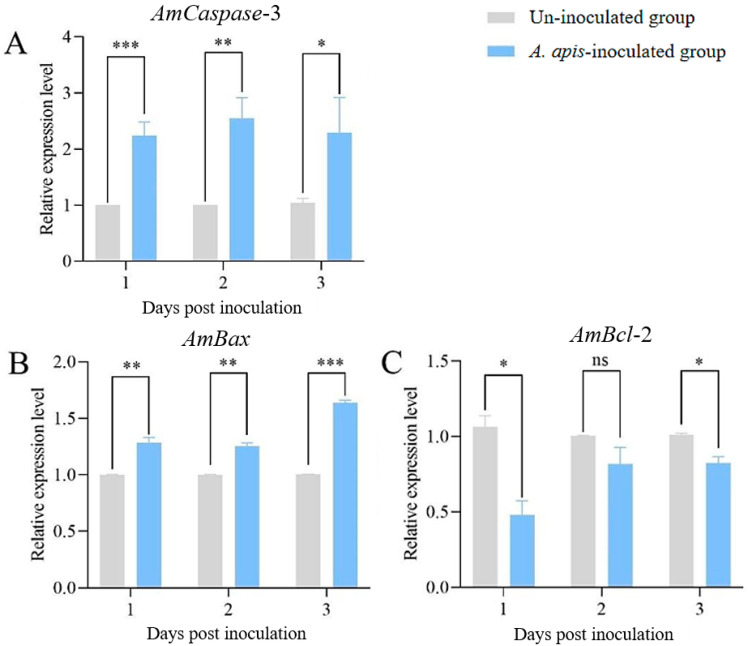
The relative expression levels of *AmCaspase*-3 (**A**), *AmBax* (**B**), and *AmBcl*-2 (**C**) in the guts of worker larvae inoculated with *A. apis*. Data are presented as mean ± SEM. Multiple *t* tests, Holm–Sidak method, *, *p* < 0.05; **, *p* < 0.01; ***, *p* < 0.001; ns, not significant.

Compared to uninoculated larval guts, the expression level of *AmCaspase*-3 showed significant upregulation (*p* < 0.01) in *A. apis*-inoculated larval guts at 1 to 3 dpi (Figure 1A). Similarly, *AmBax* expression levels were significantly elevated (*p* < 0.01) at 1–3 dpi (Figure 1B). Conversely, the significant downregulation of *AmBcl*-2 (*p* < 0.05) was observed at 4 dpi and 6 dpi (Figure 1C).

**Figure 2:**
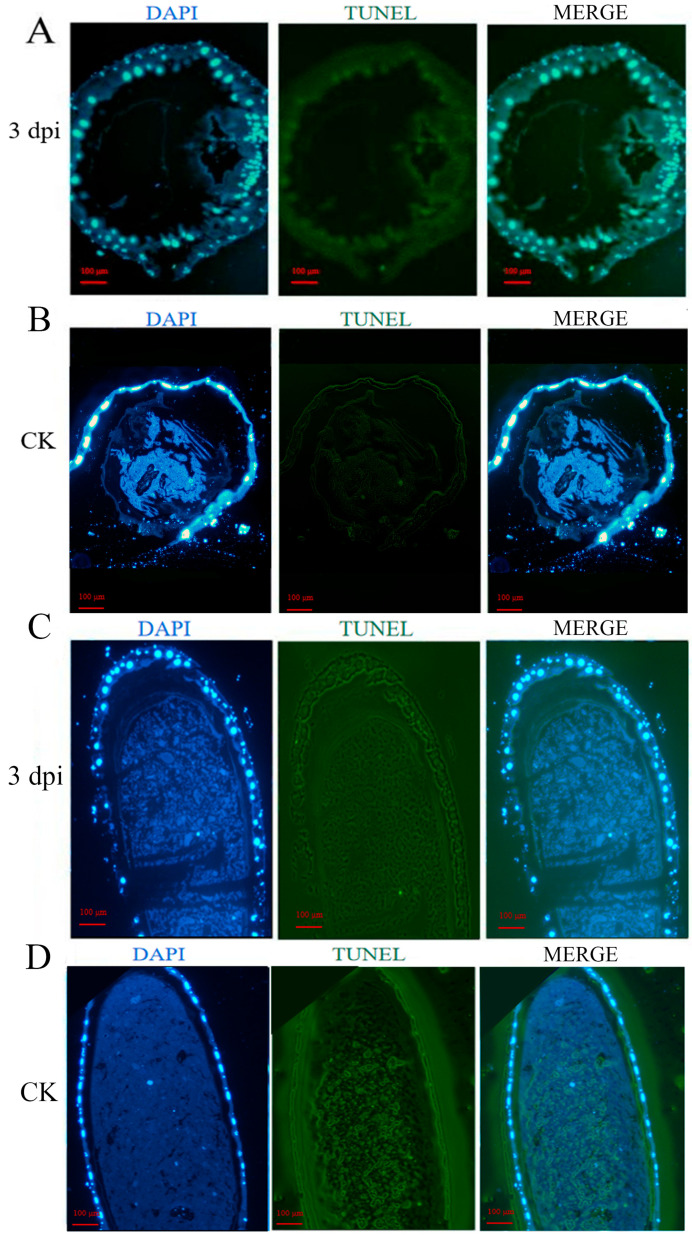
TUNEL assay of apoptosis in the gut. Cross-section of *A. apis*-infected larval gut (**A**), Cross-section of uninfected control larva (**B**), Longitudinal section of *A. apis*-infected larval gut (**C**), Longitudinal section of uninfected control larva (**D**).

TUNEL staining revealed minimal green fluorescence in uninfected larval guts, whereas strong fluorescent signals were observed in *A. apis*-infected samples. Merged images confirmed co-localization of the TUNEL signal (green) and cell nuclei (blue) in infected larval guts (Figure 2), indicating that *A. apis* infection induced significant apoptosis.

### 3.2. A. apis Affects the Survival of Worker Larvae Through the Apoptosis Process

**Figure 3:**
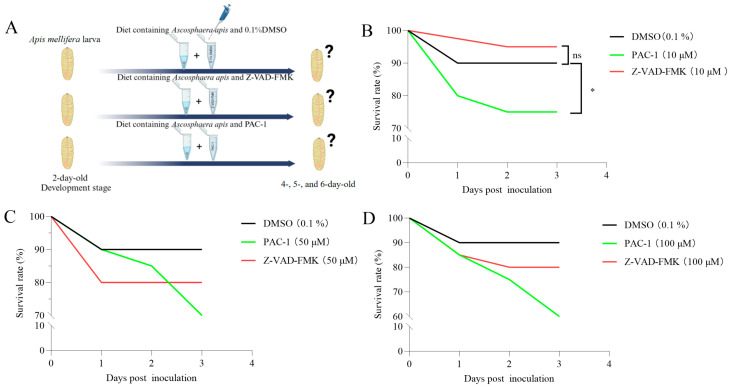
The survival rate of worker larvae fed apoptosis inhibitors and activators. (**A**) Schematic diagram of the feeding method with apoptosis inhibitors and activators. (**B**–**D**) Larval survival rates following administration of 10 μM (**B**), 50 μM (**C**), or 100 μM (**D**) activators/inhibitors. log-rank test, *, *p* < 0.05; ns, not significant.

To assess the impact of *A. apis*-induced apoptosis on worker larval survival, infected larvae were treated with the apoptosis inhibitor Z-VAD-FMK or the activator PAC-1 (Figure 3A). Treatment with the apoptosis inhibitor (10 μM) significantly improved the survival of infected larvae, whereas the apoptosis activator (10 μM) reduced survival. At higher concentrations (50 μM and 100 μM), both compounds led to a decrease in survival; however, inhibitor-treated larvae consistently exhibited higher survival rates than those receiving the activator (Figure 3C,D).

### 3.3. Inhibition and Activation of Apoptosis Affected the Expression of Apoptosis-Relevant Genes in the Infected Larval Guts and A. apis Virulence Factor Ste11-like Gene

**Figure 4:**
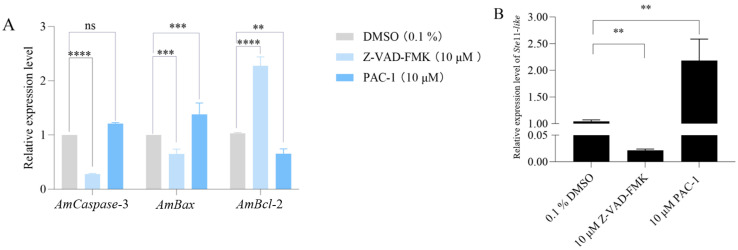
Relative expression levels of *AmCaspase*-3, *AmBax*, and *AmBcl*-2 following the inhibition and activation of apoptosis in the gut of worker larvae infected by *A. apis* (**A**), and the virulence factor *Ste*11-*like* in *A. apis* (**B**). Data are presented as mean ± SEM. Multiple *t* tests, Holm–Sidak method, **, *p* < 0.01; ***, *p* < 0.001, ****, *p* < 0.0001; ns, not significant.

RT-qPCR analysis revealed that inhibition of apoptosis in *A.Apis*-infected larvae significantly downregulated *AmCaspase*-3 (*p* < 0.001), while apoptosis activation markedly increased its expression. Similarly, *AmBax* expression decreased significantly upon apoptosis inhibition (*p* < 0.001) and increased upon activation (*p* < 0.001). In contrast, *AmBcl*-2 expression was significantly upregulated when apoptosis was inhibited (*p* < 0.001) and downregulated following apoptosis activation (*p* < 0.01) (Figure 4A). Additionally, inhibition of host apoptosis resulted in significantly reduced expression of the *A. apis Ste*11-*like* gene (*p* < 0.01), whereas apoptosis activation induced its significant upregulation (*p* < 0.01) (Figure 4B).

## 4. Discussion

The pro-apoptotic gene *Bax* acts as a molecular “switch” that triggers the host’s apoptotic defense response [21]. In contrast, the anti-apoptotic *Bcl*-2 serves as a “brake,” indicating fungal hijacking of host anti-apoptosis mechanisms for immune evasion [22]. The apoptosis executor *Caspase*-3 directly confirms completion of the apoptotic program [23]. Correlating these three biomarkers with disease phenotypes and molecular mechanisms may identify therapeutic targets for chalkbrood disease and establish a foundation for developing apoptosis-targeted control strategies (e.g., RNAi or specific inhibitors) [24]. Following the *A. apis* inoculation, the expression levels of *AmCaspase*-3 and *AmBax* in honeybee larval midguts were significantly upregulated at 1–3 dpi (Figure 1A,B), whereas the *AmBcl*-2 exhibited marked downregulation at 1 dpi and 3 dpi (Figure 1C). This coordinated dysregulation likely facilitates apoptosis, potentially benefiting fungal pathogenesis. As an anti-apoptotic regulator, the downregulation of *AmBcl*-2, a key suppressor of mitochondrial apoptosis, implies a weakening of host intrinsic mechanisms aimed at maintaining cellular integrity [25]. Concurrently, the upregulation of *AmCaspase*-3 and *AmBax* drives irreversible commitment to apoptosis. This process may be mediated by *A. apis*, potentially through fungal effectors targeting host apoptotic pathways. This mechanism aligns with strategies observed in human fungal pathogens, where virulence factors activate host caspase family proteins to promote cell death and dissemination [26]. Furthermore, sustained and overwhelming activation of executioner caspases like *Caspase*-3 by virulence factors during infection progression would ultimately lead to irreversible apoptosis.

TUNEL assays revealed intensive DNA fragmentation signals in the larval gut at 3 dpi with *A. apis*, whereas only sporadic signals were observed in the control gut (Figure 2C). The results are consistent with gene expression profiles, indicating that the *A. apis* infection induced significant apoptosis of the challenged larval gut. Notably, DAPI staining was suggestive of intact nuclear morphology, confirming that apoptosis occurred via programmed cell death rather than necrosis. This aligns with the “apoptosis induction for nutrient acquisition” strategy commonly employed by fungal pathogens [27]. *Candida albicans* secretes toxins to trigger epithelial apoptosis and disrupt host immune barriers [28]. Similarly, *A. apis* was likely to activate the host *Caspase*-3 pathway to promote intestinal epithelial apoptosis, potentially hyphal invasion through compromised tissue integrity.

Treatment with the inhibitor Z-VAD-FMK increased larval survival rates, whereas the activator PAC-1 significantly decreased survival, offering direct evidence for the dual role of apoptosis in the pathogenesis of *A. apis* (Figure 3B). On one hand, moderate apoptosis may serve as a host defense mechanism to eliminate infected cells [29]. On the other hand, excessive apoptosis may lead to loss of intestinal barrier function, accelerating pathogen dissemination [30]. A similar phenomenon was also observed in the immune responses of insects to bacterial infection. Following infection with *Bacillus bombysepticus*, the silkworm (*B. mori*) triggered comprehensive immune responses encompassing cellular immune (e.g., phagocytosis) and humoral immune (e.g., antimicrobial peptide expression). While a moderate immune response can clear the pathogen, overactivation may induce systemic inflammation or tissue damage [31]. In the present study, Z-VAD-FMK, by inhibiting *Caspase*-3 activity and reducing apoptosis levels, likely delayed intestinal epithelial damage, thereby providing the host with additional time to clear the pathogen.

Upon the inhibition of apoptosis, the expression of the *A. apis* virulence factor gene *Ste*11-*like* was significantly reduced. Conversely, activation of apoptosis led to its marked upregulation (*p* < 0.01) (Figure 4B). These results suggested that the apoptotic state of host cells may feedback-regulate pathogen virulence gene expression through an unknown signaling pathway. The Ste11 protein in fungi typically participates in the MAPK signaling pathway, regulating hyphal development and stress responses [32]. It is hypothesized that *A. apis* may sense host cell apoptosis-associated metabolic alterations (e.g., fluctuations in ATP levels or reactive oxygen species bursts), subsequently upregulating *Ste*11-*like* to enhance its fitness. A similar strategy of adapting to host-derived signals is documented in studies of human pathogens, e.g., the infection with *Bacillus anthracis* causes anthrax in humans and animals [33,34]. During the infection process of host macrophages, *B. anthracis* sensed and rapidly adapted to the intracellular environment and modulated its metabolic pathways, including energy metabolism and cofactor biosynthesis, to enhance its intracellular survival [35]. *Mycobacterium tuberculosis* subverted the pyroptosis by hijacking the host ubiquitin system to remodel membrane lipid homeostasis [36]. This ability of pathogens to sense and adapt to host-derived cues, akin to *B. anthracis* and *M. tuberculosis* in macrophages, underscores the sophistication of the feedback regulation we observed between larval apoptosis and *A. apis* virulence.

## 5. Conclusions

**Figure 5:**
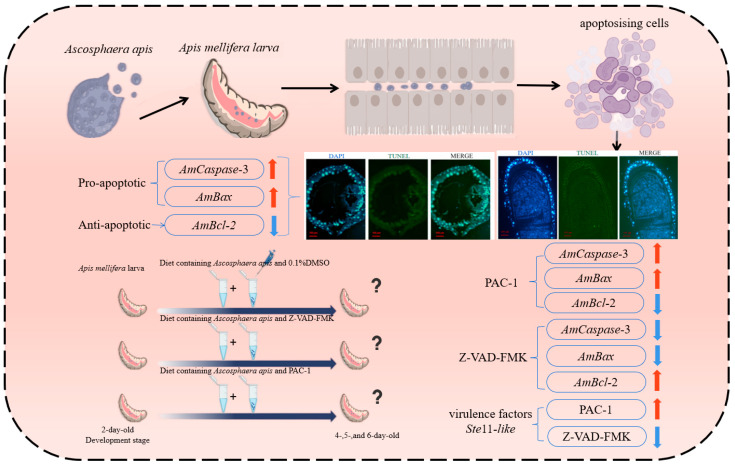
Schematic diagram depicting the apoptotic status of host gut cells, expression changes in apoptosis-related genes, and expression patterns of fungal virulence factors in the gut of *A. apis*-infected honeybee larvae during pathogenesis. The upward red arrow indicates a upregulated expression of the gene; the downward blue arrow indicates a downregulated expression of the gene.

*A. apis* employs multi-level modulation of the host apoptosis pathway (Caspase-3/Bcl-2/Bax axis) to adapt to the infection microenvironment, while demonstrating a dynamic interplay between host apoptotic status and pathogen virulence factor gene expression. This work lays the groundwork for the theoretical basis of targeted control strategies focusing on the apoptosis-virulence interplay node (Figure 5).

## Figures and Tables

**Table 1:** Primer information.

Gene	Sequence (5′-3′)	Purpose
*GAPDH*	F: CACCTTCTGCAAAATTATGGCG R: ACCTTTGCCAAGTCTAACTGTTAA	Reference gene for RT-qPCR
*Caspase*-3	F: ACCTGATCACTCGTTCCACT R: AGCAAGATGGAAAACGTGTGT	RT-qPCR
*Bcl*-2	F: GAATATGTAGCCCGCATCTTTT R: CTTTGTTGATTAGACTTGCCGA	
*Bax*	F: CTGCTGCAGGAGTTTACATTCA R: AAGTAAAGGACCCAGGCCAA	
*Actin apis*	F: CATGATTGGTATGGGTCAG R: CGTTGAAGGTCTCGAAGAC	Reference gene for detection of virulence factors in *A. apis*
*Ste*11*-like*	F: GGGAAGATTGCCAGGCC R: CACTTGTAGTCCGGATG	Detection of virulence factors in *A. apis*

## Data Availability

All the data are contained within the article.
